# Artistry and Aesthetics in Breast Reconstruction: Raising the Bar

**DOI:** 10.3390/jcm12134459

**Published:** 2023-07-03

**Authors:** Pietro Giovanni di Summa, Gianluca Sapino

**Affiliations:** Department of Plastic and Hand Surgery, Centre Hospitalier Universitaire Vaudois (CHUV), 1011 Lausanne, Switzerland

Breast reconstruction is a critical component of breast cancer treatment for many women who undergo mastectomy. It aims to restore the appearance of breasts and to improve patients’ quality of life. In recent years, significant advancements in breast reconstruction techniques have been made, allowing for more natural-looking and aesthetically appealing results. It has been exciting for plastic surgeons to witness these developments firsthand and offer the latest options for breast reconstruction to patients.

As a result of surgical innovation and experience throughout the years, a satisfactory outcome in free flap breast reconstruction is no longer just a simple flap survival rate. Nowadays, surgeons are focusing their efforts on giving patients the best aesthetic results while performing breast reconstruction. Autologous reconstruction is considered a standard of care in many hospitals and countries, expanding prospects when implant-based reconstruction fails in radiotherapy settings, and benefitting all patients who desire a more natural solution and who present localized excess tissue. Nowadays, advancements in microsurgical practice allow the right flap to be chosen for the right patient, transforming the potential donor site morbidity, due to flap harvest, into a clinical advantage for the patient that can improve body shape.

Particular attention is reserved for donor site management. Developments in aesthetic procedures (such as abdominoplasty or thigh lift) are routinely applied when dealing with a flap donor site, resulting in improved contour as a result of pre-operative liposuction and well-hidden scars. Careful pre-operative planning using an angio-CT scan becomes mandatory in order to identify a suitable perforator, and is ideally lower than the umbilicus, forming a low abdominal scar. Some groups even advocated for the routine use of a superficial vein to supercharge the flap when the flap is harvested on a non-dominant perforator and to reduce the risk of jeopardizing the vascularization. Even if such procedures need to be attentively planned, by weighing various risks and benefits, they demonstrate a global trend towards the maximization of aesthetic outcomes [1].

Furthermore, in abdominal-based free flaps, interest in preserving abdominal fascia and muscle tissue to improve functionality has increased. In large-volume microsurgical units with high-standard microsurgical practices, limited fascia incision, robotic harvest, and pedicle disassembly (abdominal perforator exchange, APEX) are all smart solutions to improve functional and esthetic outcomes [2,3].

If a single breast following a skin sparing mastectomy can often be reconstructed with a single flap, more tissue is sometimes required to guarantee the most natural shape and volume. In this sense, stacked and conjoined flaps are gaining popularity due to microsurgical advancements. The benefit of stacked or conjoined flaps is that, when used appropriately, they nearly always provide all required aspects of breast reconstruction. A naturally aesthetically appealing breast must have a stable foundation on the chest wall, stable volume in all quadrants (providing three-dimensional projection), and adequate skin to allow natural ptosis. The use of multiple flaps for a single breast allows for a tailored approach based on patient-centric factors and provides all aspects required for a natural breast reconstruction.

Importantly, new frontiers in microsurgery are not adequately paralleled by innovations in oncologic surgery. Conservative mastectomies (skin-sparing, nipple-sparing, and skin-reducing mastectomies) are accepted as new techniques that improve aesthetic results and patient quality of life. Such mastectomies combine the oncological advantage of a complete glandular excision with the optimal cosmetic result of preserving the skin envelope and, wherever possible, the nipple–areola complex. Immediate breast reconstruction (IBR) using free flaps after nipple-sparing and skin-reducing mastectomies can provide natural-looking outcomes in a one-step surgical approach, providing several advantages for both the patient and the healthcare system, in a time- and cost-effective way. Indeed, studies show no negative impact on recurrence or patient survival, but better aesthetic outcome, less psychological distress, and lower treatment costs in IBR [4]. Finally, in the last decade, lymphatic surgery advancements have become extremely popular and have also affected the breast reconstruction journey. Abdominal flaps can be harvested, including abdominal lymph nodes, which can be transferred to the axillary fold to treat upper-limb lymphedema. Combined breast and lymphatic procedures have helped to significantly improve quality of life, with a reduced need for physiotherapy and compressive garments [5].

Implant-based breast reconstruction (IBR) has also experienced important advancements, despite becoming less popular after the association between anaplastic large cell lymphoma (ALCL) and macro-textured implants [6]. Round implants have become more popular for breast reconstruction; however, these devices may have issues around implant displacement and lower pole tissue stretch. This is due to the thin capsule formed around the implant and the lack of tissue ingrowth, thereby not providing stability in the pocket. Proper patient selection and planning, the identification of risk factors, correct implant size selection, and meticulous surgical techniques are of a paramount importance in order to significantly minimize the incidence of mentioned complications [7].

Moreover, IBR has been traditionally performed by placing the implant in a submuscular pocket beneath the pectoralis major muscle. Lastly, pre-pectoral breast reconstruction has gained popularity due to numerous benefits in properly selected patients, as well as the pre-pectoral plane, which is also the most anatomically correct implant position to reconstruct the breast. Compared with subpectoral implant reconstruction, pre-pectoral reconstruction helps to preserve the pectoralis major muscle in its native position, resulting in decreased pain, no animation deformity, and an improved arm range of motion/strength. Although pre-pectoral reconstruction is safe and effective, the implant sits closer to the mastectomy skin flap. Acellular dermal matrices may play a critical role, allowing for the precise control of the breast envelope and providing long-term implant support [8]. In addition, polyurethan implants may be used with no matrices, reducing the costs of reconstructions. The micro-polyurethane foam-coated shell surface, for instance, improves implant stability over time. Careful patient selection and intraoperative mastectomy flap evaluation is critical in order to obtain optimal results with pre-pectoral breast reconstruction. Pre-operative indocyanine green angiography has largely helped surgeons to evaluate the mastectomy flap viability, improving patient selection for pre-pectoral breast reconstruction while reducing risks of complications (skin necrosis, among others) [9]. One downside to pre-pectoral implant placement compared to at least an upper pole covered by the pectoral muscle is an increased incidence of rippling. Rippling can be masked with fat grafting; thus, more and more groups have advocated for primary fat grafting at the time of implant placement following the concept of “hybrid breast augmentation” [10].

In conclusion, breast reconstruction has come a long way in recent years, as a result of advances in microsurgery, mastectomy techniques, and pre-pectoral breast reconstruction. Such improvements have raised the bar for breast reconstruction, aiming for more natural-looking and aesthetically pleasing results than ever before. Improved knowledge and skills have become essential in order to offer our patients the best possible outcomes, possibly exceeding their expectations.
